# Effect of PCI on ophthalmic artery hemodynamics in patients with acute coronary syndrome

**DOI:** 10.3389/fmed.2024.1367900

**Published:** 2024-03-04

**Authors:** Wen-long Liu, Lan-ting Wu, Jia-lin Wang, Jiao Sun, Xue-ru Cheng, Zhuo-hua Zhou, Jia-xin Guan, Yan-ling Wang, Zhao-yang Meng

**Affiliations:** Department of Ophthalmology, Beijing Friendship Hospital, Capital Medical University, Beijing, China

**Keywords:** percutaneous coronary intervention, ophthalmic artery, computational fluid dynamics, hemodynamic numerical simulation, three-dimensional reconstruction

## Abstract

**Purpose:**

We aimed to explore the effects of percutaneous coronary intervention (PCI) on the ophthalmic artery (OA) hemodynamics in patients with acute coronary syndrome (ACS).

**Methods:**

A total of 73 participants (Group0: healthy controls, Group1: Patients with ACS underwent PCI < 3 months, Group2: Patients with ACS underwent PCI ≥ 3 months) were enrolled. Computed tomographic angiography images were used to construct three-dimensional models of participants' OAs. Numerical simulations based on computational fluid dynamics were used to acquire hemodynamic parameters.

**Results:**

The angle between the OA and internal carotid artery in Group2 was significantly larger compared with Group0 and Group1 (*P* = 0.003 and *P* = 0.044). Hemodynamic simulation showed a significantly slower OA blood velocity in Group1 than in the control (*P* < 0.001) and Group2 (*P* = 0.033). Lower wall shear stress was found in Group1 than that in control (*P* = 0.040). Patients after PCI had a higher wall pressure than healthy controls (*P* = 0.012 and *P* = 0.004). Mass flow ratios were decreased in Group1 and Group2 (*P* = 0.021 and *P* = 0.002). The hemodynamic parameters of OA were correlated with several clinical indicators.

**Conclusions:**

The OA blood flow velocity of patients with ACS after PCI initially slowed down, which increased the risk of plaque formation, and then showed an increasing trend. There was a correlation between OA hemodynamic parameters and clinical indexes related to cardiac stress. Ischemia-reperfusion injury and changes in blood flow status after PCI may affect OA morphology and hemodynamics, leading to ocular lesions.

**Trial registration:**

ChiCTR2100050428.

## Introduction

Cardiovascular disease is the leading cause of death worldwide. Despite the initial results, the treatment of up to 197 million patients with ischemic heart disease worldwide still faces significant challenges (1). Percutaneous coronary intervention (PCI) is currently an important treatment for myocardial revascularization in patients with acute coronary syndrome (ACS), reducing the risk of death and improving the long-term prognosis. Advances in technology and development of antiplatelet therapy are increasing the safety of the procedure (2). However, significant complications can still occur during and after PCI that may be related to the puncture site, the oral catheterization of the coronary artery, or the intervention itself. Moreover, myocardial ischemia-reperfusion injury (IRI) after PCI should not be ignored (3). Different organs, including the kidney, brain, eyes, and gastrointestinal system, may be targets of thromboembolic events after PCI (4). Since 1985, cases of ocular complications after PCI have been reported from around the world (5–7). Ophthalmic complications of PCI include a wide range of clinical manifestations, from transient visual impairment to permanent and devastating conditions. Most cases describe retinal artery occlusion (RAO) after PCI. The incidence of new retinal embolism after PCI was 6.33% (8). Therefore, it is of great significance to explore the changes of ocular blood flow after PCI for the prevention and treatment of ocular diseases such as RAO.

Our previous study found that ophthalmic artery (OA) can reflect changes in ocular blood supply earlier than retinal vessels (9). Using computational fluid dynamics (CFD), we found that OA in patients with ACS had a slower blood flow velocity (10). The numerical simulation technology based on CFD has solved the difficult problem of OA observation and measurement, and provided an effective means for exploring the relationship between OA and ischemic heart disease. However, there is a lack of research on retrobulbar blood flow changes after PCI. Therefore, the purpose of this study was to investigate the effects of PCI on the OA hemodynamics in patients with ACS.

## Methods

### Study design and participants

This study (ChiCTR2100050428) included patients with ACS who underwent head and neck computed tomographic angiography (CTA) examination after PCI at Beijing Friendship Hospital between September 2021 and January 2023, as well as healthy controls (HCs) who received CTA for other reasons. The study protocol was approved by the local ethics committee of the Beijing Friendship Hospital (2020-P2-008-01) and conformed to the tenets of the Declaration of Helsinki. All participants provided written informed consent. Three groups were defined, prior to recruitment: HCs (Group 0), patients with ACS underwent PCI < 3 months (Group 1), and patients with ACS underwent PCI ≥ 3 months (Group 2).

A detailed ophthalmic examination was performed on each participant, including best-corrected visual acuity, intraocular pressure and slit-lamp examination. The slit-lamp examination was performed by two experienced ophthalmologists. Patients with significant ocular lesions such as glaucoma, orbital space-occupying diseases, and optic neuritis, as well as those caused by systemic diseases such as diabetic retinopathy, were excluded. The electronic medical records were collected retrospectively to record general information, laboratory parameters, echocardiogram results, coronary angiography results, and concomitant medications.

### Ophthalmic artery computational fluid dynamics simulation

Based on our previous research method (10), the original head and neck CTA images of all participants were obtained, and the three-dimensional OA models were reconstructed. Import the CTA DICOM image into Mimics 21.0 (Materialize, Ann Arbor, MI, USA). An image segmentation technique was used to reconstruct one of the OAs visible on the CTA image for each participant. Manually edit model boundaries to eliminate adjacent interference structures. A solid blood vessel model was obtained after smoothing the surface of the model in Geomagic Studio 14.0 (3D Systems, Rock Hill, SC, USA).

Based on CFD, the finite-volume method was adopted and ANSYS Fluent 15.0 (ANSYS, Inc., Canonsburg, PA, USA) was used for hemodynamic numerical simulation. The blood vessels were assumed to be rigid and non-slipped, and the simulated blood was considered to be a steady-state, laminar, incompressible Newtonian fluid. The governing equations for the numerical simulation were the Navier-Stokes equation and mass conservation equation (Equations 1, 2):


(1) $$\begin{array}{l} \rho \left(\vec{u}\cdot \nabla \right)\vec{u}+\nabla p-\mu \Delta \vec{u}=0 \end{array}$$

(2) $$\begin{array}{l} \nabla \cdot \vec{u}=0 \end{array}$$

In the formula, $\vec{u}$ represents the velocity vector, *p* is the pressure, ρ is the blood density, and μ is blood viscosity. The blood viscosity and density were set to 3.5 × 10^−3^ kg/ms and 1,050 kg/m^3^, respectively. Based on the literature (11), we adopted a systolic and diastolic mean flow velocity of 0.34 m/s as the inlet velocity (velocity of the internal carotid artery [ICA] siphon). All models were set to the same boundary conditions.

### Quantitative assessment

We measured the morphological data of the OA models. The centerline of each model was generated to obtain the best-fit diameter of the initial OA and the angle between the OA and ICA centerline. The initial OA was defined as the region where OA originates from ICA. Two experienced ophthalmologists collected all the data.

After successful simulation, the OA hemodynamic data were obtained by using the Ansys Fluent post-processing software. The blood flow velocity, wall shear stress (WSS), and initial OA pressure were obtained quantitatively. The mass flow of the OA and ICA in flux reports was obtained. Additionally, the mass flow ratio, defined as the mass flow of the OA accounted for ipsilateral ICA mass flow, was calculated.

### Statistical analysis

Statistical analyses were performed using SPSS Statistics 26.0 (IBM, Armonk, NY, USA). The Shapiro-Wilk test was used to test the normality of the variables. Data for normal distribution are expressed as mean ± standard deviation, and descriptive data for non-normal distribution are expressed as median (25–75%). In the multi-group comparison, one-way ANOVA with Bonferroni correction was used for continuous variables with normal distribution, and Kruskal-Wallis *H* test was used for variables with non-normal distribution. Depending on normality, comparisons between the two groups were made using either the *t*-test or Mann-Whitney *U* test. Categorical variables were expressed as numbers and percentages and analyzed using χ^2^ or Fisher's exact tests, as appropriate. Pearson's correlation coefficient and linear regression were used to determine correlations between continuous variables. Non-normally distributed variables were converted into natural logarithms.

## Results

### Clinical characteristics

**Figure 1:**
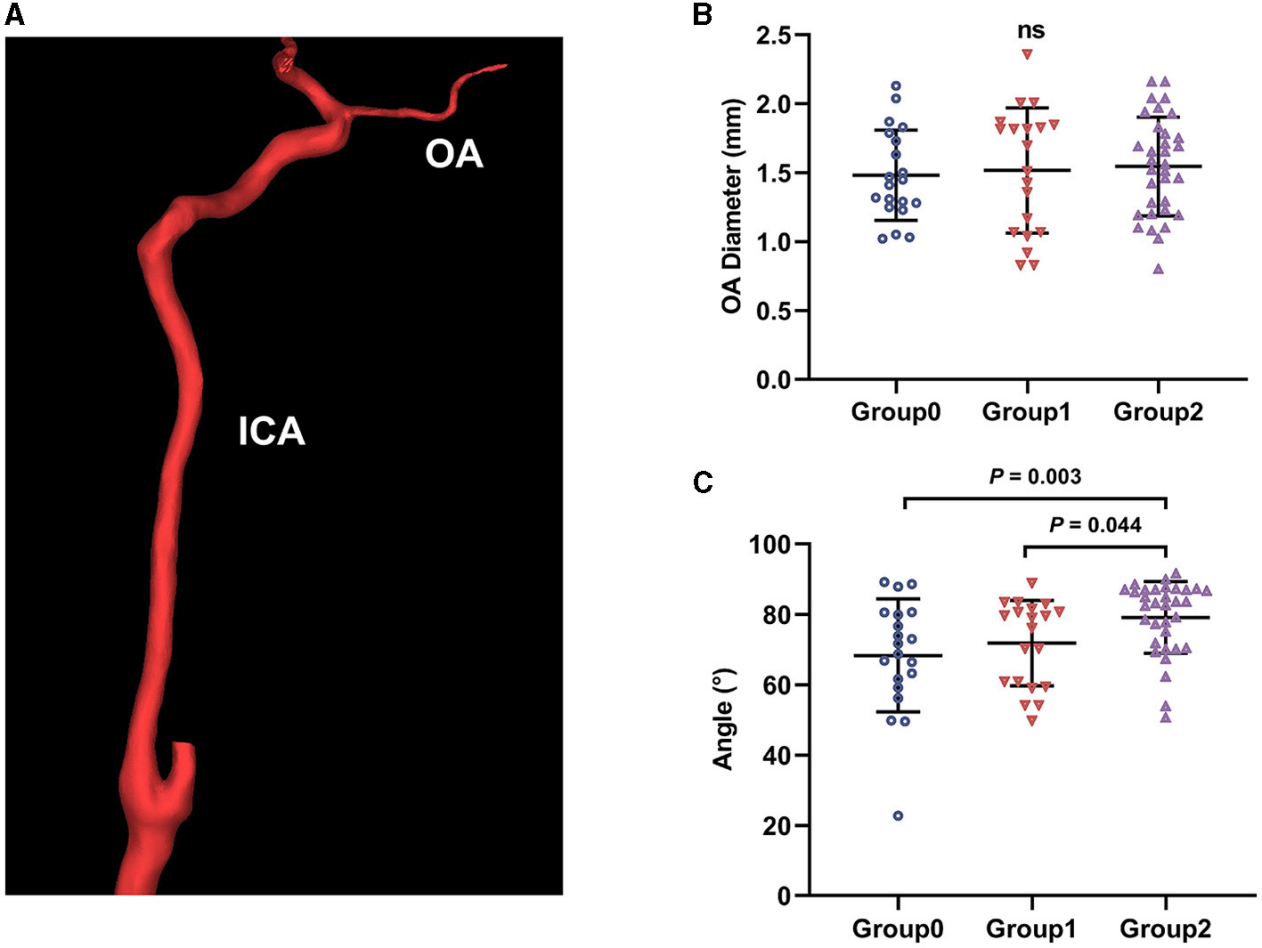
Ophthalmic artery (OA) three-dimensional reconstruction and morphological measurement. **(A)** The reconstructed OA model; ICA, internal carotid artery. **(B)** The initial OA diameter comparison between groups; ns, not significant. **(C)** Angle between OA and ICA comparison between groups.

In total, 73 OA models were reconstructed (Figure 1A). Table 1 shows the clinical characteristics of the 73 participants. The groups were matched for age (*P* = 0.084), sex (*P* = 0.173) and type of ACS (*P* = 0.721, *P* = 0.801, *P* = 0.591, respectively). A higher proportion of patients with ACS had diabetes, and dyslipidemia (*P* = 0.001 and *P* = 0.011, respectively). There were no differences in peripheral arterial disease (*P* = 0.577), history of ischemic stroke (*P* = 0.092), family history of coronary atherosclerotic heart disease (*P* = 0.679), a current smoking status or hypertension (*P* = 0.027, *P* = 0.021 not significant after Bonferroni correction). The clinical, laboratory, echocardiographic, and medication details of the patients after PCI are shown in Table 2.

**Table 1:** Baseline characteristics of the participants.

**Variables**	**Group0**	**Group1**	**Group2**	***P* value**
	**(*n* = 20)**	**(*n* = 20)**	**(*n* = 33)**	
Age (y), mean ± SD	61.80 ± 4.35	62.70 ± 4.08	64.94 ± 6.18	0.084
Female sex, *n* (%)	8 (40)	4 (20)	6 (18)	0.173
STEMI, *n* (%)	-	4 (20)	8 (24)	0.721
NSTEMI, *n* (%)	-	6 (30)	11 (33)	0.801
UA, *n* (%)	-	10 (50)	14 (42)	0.591
Current smoking, *n* (%)	8 (40)	16 (80)	22 (67)	0.027
Hypertension, *n* (%)	12 (60)	18 (90)	29 (88)	0.021
Diabetes mellitus, *n* (%)	4 (20)	14 (70)	22 (67)	**0.001**
Dyslipidaemia, *n* (%)	10 (50)	18 (90)	26 (79)	**0.011**
PAD, *n* (%)	4 (20)	6 (30)	11 (33)	0.577
History of ischemic stroke, *n* (%)	2 (10)	8 (40)	10 (30)	0.092
Family history of CAD, *n* (%)	5 (25)	6 (30)	12 (36)	0.679

STEMI, ST-segment elevation myocardial infarction; NSTEMI, non-STEMI; UA, unstable angina; PAD, peripheral arterial disease; CAD, coronary atherosclerotic heart disease. Bonferroni correction was used for multiple comparisons. Bold values are significant.

**Table 2:** Baseline characteristics of patients after PCI.

**Variables**	**Group1**	**Group2**	***P* value**
	**(*n* = 20)**	**(*n* = 33)**	
**Clinical characteristics**			
BMI (kg/m^2^), mean ± SD	24.76 ± 2.07	26.53 ± 2.50	**0.010**
DAC (cm), mean ± SD	92.20 ± 8.57	91.64 ± 9.97	0.834
Heart rate (bpm), mean ± SD	74.10 ± 10.86	68.58 ± 13.28	0.123
Systolic BP (mmHg), mean ± SD	140.90 ± 23.19	136.97 ± 23.39	0.555
Diastolic BP (mmHg), mean ± SD	80.40 ± 14.84	79.12 ± 19.10	0.799
**Laboratory parameters**			
TnI (ng/mL), median (IQR 25%−75%)	0.59 (0.06–3.77)	0.02 (0.003–0.54)	0.061
TnT (ng/mL), median (IQR 25%−75%)	0.07 (0.03–0.78)	0.18 (0.01–0.12)	0.133
CK (U/L), median (IQR 25%−75%)	61.50 (56.00–122.50)	95.00 (59.00–250.50)	0.368
CK–MB (ng/mL), median (IQR 25%−75%)	1.80 (1.45–4.63)	1.2 (0.83–4.60)	0.349
LDH (U/L), median (IQR 25%−75%)	175.00 (154.00–186.75)	194.00 (150.00–222.00)	0.826
NT–proBNP (pg/mL), median (IQR 25%−75%)	855.00 (137.00–1438.25)	229.50 (106.00–848.75)	0.106
Scr (μmol/L), mean ± SD	72.67 ± 10.65	67.11 ± 10.07	0.062
FBG (mmol/L), median (IQR 25%−75%)	6.50 (5.90–9.58)	6.35 (6.03–9.18)	0.732
HBA1c (%), median (IQR 25%−75%)	7.96 (6.03–8.65)	6.60 (6.10–7.65)	0.293
TC (mmol/L), mean ± SD	3.84 ± 0.97	3.75 ± 1.01	0.747
TG (mmol/L), mean ± SD	1.51 ± 0.60	1.32 ± 0.60	0.274
HDL (mmol/L), mean ± SD	1.15 ± 0.59	1.11 ± 0.27	0.723
LDL (mmol/L), mean ± SD	2.01 ± 0.81	2.06 ± 0.77	0.831
Sodium (mmol/L), mean ± SD	140.77 ± 2.36	139.61 ± 2.00	0.075
Potassium (mmol/L), mean ± SD	4.01 ± 0.43	3.98 ± 0.32	0.729
TyG index, mean ± SD	7.43 ± 0.62	7.29 ± 0.58	0.447
**Echocardiography, mean** ±**SD**			
LVEF (%)	60.90 ± 9.78	63.22 ± 7.16	0.324
E/A	0.77 ± 0.22	0.83 ± 0.23	0.388
Cardiac index (L/min/m^2^)	2.65 ± 0.51	2.86 ± 0.60	0.206
**Concomitant medication**, ***n*** **(%)**			
Statin	16 (80)	31 (94)	0.184
Aspirin	16 (80)	27 (82)	0.870
Clopidogrel/Ticagrelor	18 (90)	21 (64)	**0.035**
ACE inhibitor/ARB	2 (10)	25 (76)	**< 0.001**
Beta blocker	12 (60)	21 (64)	0.791
Calcium channel blocker	6 (30)	13 (39)	0.489
Insulin	2 (10)	4 (12)	0.813

BMI, body mass index; BP, blood pressure; TnI, troponin I; IQR, interquartile range; TnT, troponin T; CK, creatine kinase; CK-MB, creatine kinase isoenzyme-MB; LDH, lactate dehydrogenase; NT-proBNP, N-terminal pro-B-type natriuretic peptide; Scr, serum creatinine; FBG, fasting blood glucose; HBA1c, hemoglobin A1c; TC, total cholesterol; TG, triacylglycerol; HDL, high-density protein; LDL, low-density protein; TyG, triglyceride glucose index, calculated as the ln [fasting triglycerides (mg/dL) × fasting plasma glucose (mg/dL)/2]; LVEF, left-ventricular ejection fraction; E/A, ratio of early to late transmitral flow velocity; ACE, angiotensin-converting enzyme; ARB, angiotensin receptor blocker. P < 0.05 is significant (bold values).

Coronary artery lesions of patients were assessed through angiography: two patients in the Group1 had a single vessel lesion (left anterior descending artery [LAD] involved), two patients had the left main stem and three vessel lesions (left main coronary artery, LAD, left circumflex artery [LCX], and right coronary artery [RCA] involved); three patients in the Group2 had single-vessel lesions (LAD or RCA involved). In addition, all the other patients had multiple vessel lesions (involving the LAD, LCX, and RCA). The mean time after PCI of Group1 was 10.30 ± 9.46 days, and that of Group 2 was 777.10 ± 525.21 days.

### Morphological and hemodynamic changes

We obtained the initial OA morphological data of all participants by measuring the model. The mean diameters of the initial OA were 1.48±0.33 mm, 1.52±0.45 mm, and 1.54±0.36 mm for Group0, Group1, and Group2, respectively. No significant difference was found in the diameter (*P* = 0.838, Figure 1B). The angles between the OA and ICA were 68.34 ± 16.04°, 71.86 ± 12.16°, and 79.16 ± 10.23° in these groups, respectively. The angles of Group2 were greater than that of Group0 and Group1 (*P* = 0.003 and *P* = 0.044, Figure 1C).

**Figure 2:**
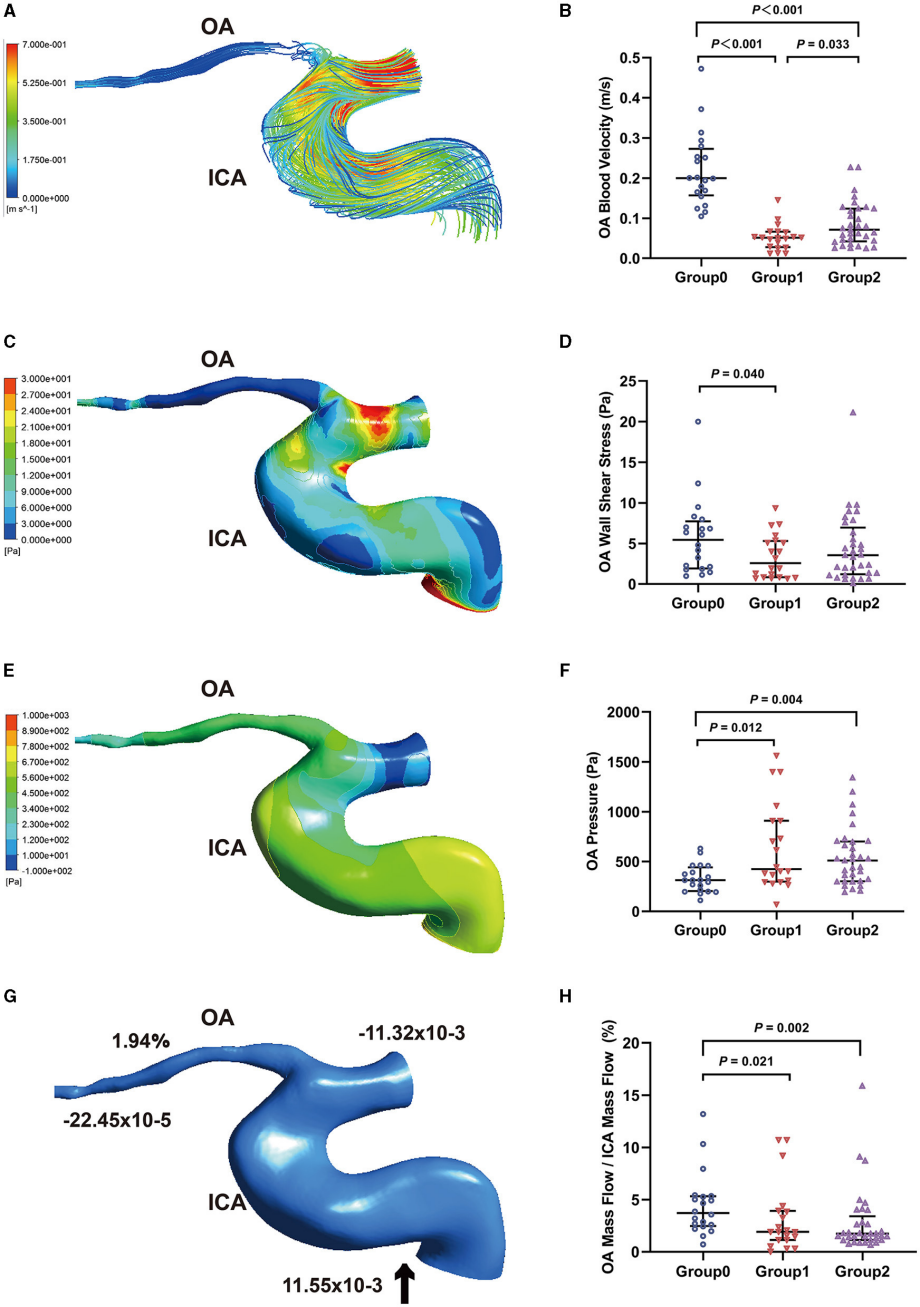
Hemodynamics characteristics of the ophthalmic artery (OA). **(A)** The streamline based on OA blood velocity. **(B)** Comparison of OA blood velocity. **(C)** The wall shear stress contour of OA. **(D)** Comparison of OA wall shear stress. **(E)** The pressure contour of OA **(F)** Comparison of OA pressure. **(G)** Mass flow (kg/s) and mass flow ratio of OA to ipsilateral internal carotid artery (ICA) (%). **(H)** Comparison of mass flow ratio of OA to ICA (%); Inlet (+), outlet (–), Blood flow direction (black arrow).

The streamline charts of each OA model were drawn according to the CFD numerical simulation results (Figure 2A). The colors in the streamlined chart indicate blood flow velocity. The closer the streamline is to red, the higher the speed. Through quantitative measurement, the initial OA blood flow velocity was 0.20 m/s (0.16–0.27 m/s), 0.05 m/s (0.03–0.07 m/s) and 0.07 m/s (0.04–0.12 m/s) in Group0, Group1 and Group2, respectively. The blood flow velocities of the OA in all disease groups were lower than those of the control group (*P* < 0.001, Figure 2B). Moreover, the Group1 had slower OA blood velocities than Group2 (*P* = 0.033).

Figure 2C shows the contour charts of the WSS. The OA WSS of the three groups were 5.44 Pa (1.93–7.72 Pa), 2.57 Pa (0.85–5.31 Pa), and 3.55 Pa (1.21–6.96 Pa), respectively. The WSS of the initial OA in Group1 was significantly lower than that in Group0 (*P* = 0.040, Figure 2D). The pressure of the initial OA (Figure 2E) was 313.20 Pa (204.25–441.47 Pa), 424.53 Pa (298.78–910.08 Pa), and 510.32 Pa (301.26–700.60 Pa) in Group0, Group1, and Group2, respectively. The Group1 and Group2 had a higher OA pressure than the control group (*P* = 0.012 and *P* = 0.004, Figure 2F).

Through calculation, we obtained mass flow data for each OA model (Figure 2G). The mass flow ratios of the OA to the ipsilateral ICA were 3.72% (2.47–5.32%), 1.91% (1.12–3.93%), and 1.74% (1.13–3.41%), respectively. The mass flow ratios in Group1 and Group2 were lower than those in Group0 (*P* = 0.021 and *P* = 0.002, Figure 2H).

### Correlation between OA characteristics and clinical parameters

Table 3 shows the correlations between OA characteristics and clinical parameters. The pressure of the initial OA was positively correlated with the ratio of early to late transmitral flow velocity (*r* = 0.306, *P* = 0.029), troponin I (TnI, *r* = 0.369, *P* = 0.006), troponin T (TnT, *r* = 0.318, *P* = 0.020), and N-terminal pro-B-type natriuretic peptide (NT-proBNP *, r* = 0.550, *P* < 0.001). The pressure of the initial OA was negatively correlated with high-density protein (*r* = −0.317, *P* = 0.021). The mass flow ratios of the OA to the ipsilateral ICA were positively correlated with TnT (*r* = 0.451, *P* = 0.001), hemoglobin A1c levels (*r* = 0.297, *P* = 0.043), potassium (*r* = 0.372, *P* = 0.006). In contrast, it was negatively correlated with sodium (*r* = −0.290, *P* = 0.035).

**Table 3:** Association between OA characteristics and clinical parameters.

**Variables**	**ln (pressure)**		**ln (mass flow ratio)**	
	**Correlation coefficient (r)**	***P*** **value**	**Correlation coefficient (r)**	***P*** **value**
BMI (kg/m^2^)	−0.256	0.065	−0.029	0.835
DAC (cm)	−0.014	0.921	0.043	0.761
Heart rate (bpm)	−0.163	0.245	0.239	0.084
Systolic BP (mmHg)	−0.112	0.425	0.052	0.711
Diastolic BP (mmHg)	−0.203	0.145	0.061	0.666
LVEF (%)	−0.299	0.099	−0.182	0.191
E/A	0.306	**0.029**	0.267	0.058
Cardiac index (L/min/m^2^)	−0.071	0.629	0.191	0.189
ln (TnI) (ng/mL)	0.369	**0.006**	0.217	0.119
ln (TnT) (ng/mL)	0.318	**0.020**	0.451	**0.001**
ln (CK) (U/L)	0.237	0.087	0.184	0.186
ln (CK–MB) (ng/mL)	0.213	0.125	0.174	0.214
ln (LDH) (U/L)	−0.160	0.251	0.154	0.270
ln (NT–proBNP) (pg/mL)	0.550	**< 0.001**	0.245	0.077
Scr (μmol/L)	−0.034	0.808	0.111	0.430
ln (HBA1c) (%)	−0.163	0.274	0.297	**0.043**
ln (FBG) (mmol/L)	−0.036	0.815	0.261	0.087
TC (mmol/L)	0.037	0.793	−0.035	0.804
TG (mmol/L)	0.000	0.998	0.039	0.784
HDL (mmol/L)	−0.317	**0.021**	−0.189	0.175
LDL (mmol/L)	0.167	0.233	0.072	0.609
Sodium (mmol/L)	−0.027	0.846	−0.290	**0.035**
Potassium (mmol/L)	−0.036	0.800	0.372	**0.006**
TyG index	0.036	0.819	0.165	0.284

Ln, natural log of the variable; BMI, body mass index; BP, blood pressure; LVEF, left-ventricular ejection fraction; E/A, ratio of early to late transmitral flow velocity; TnI, troponin I; TnT, troponin T; CK, creatine kinase; CK-MB, creatine kinase isoenzyme-MB; LDH, lactate dehydrogenase; NT-proBNP, N-terminal pro-B-type natriuretic peptide; Scr, serum creatinine; FBG, fasting blood glucose; HBA1c, hemoglobin A1c; TC, total cholesterol; TG, triacylglycerol; HDL, high-density protein; LDL, low-density protein; TyG, triglyceride glucose index, calculated as the ln [fasting triglycerides (mg/dL) × fasting plasma glucose (mg/dL)/2]. P < 0.05 is significant (bold values).

## Discussion

In this study, the morphological and hemodynamic changes of the OA in patients with ACS after PCI were observed using CFD. As reported in the literature, ocular complications after PCI include retinal complications and neuro-ophthalmic complications (8, 12). Retinal complications range from asymptomatic cotton wool spots and superficial hemorrhages to severe retinal thromboembolic events with vision loss. Atherosclerotic plaque, clots at the tip of the catheter, and foreign objects on the catheter or guide wire can all lead to a serious thromboembolic event from the heart to the eye (8). Neuro-ophthalmic complications of PCI may be caused by thromboembolic events in several important nuclei of ocular motility (13, 14).

Although reperfusion of ischemic myocardium is beneficial for improving cardiac function, delayed reperfusion is known to cause impaired recovery of contractile activity, induce arrhythmia, enhance metabolic defects, and produce structural damage to cardiomyocytes in the heart (3, 15). These abnormalities due to reperfusion of the ischemic heart are termed as IRI. The mechanism of myocardial IRI is related to a variety of factors. So far, studies have mainly focused on oxidative stress, inflammation, calcium overload, energy metabolism disorders, pyroptosis and ferroptosis (16–21). In addition to the local adverse effects on myocardium, myocardial IRI induces distant organ injury. It has been reported that myocardial IRI produces proinflammatory cytokines such as tumor necrosis factor-α (TNF-α), interleukin-1 (IL-1), and interleukin-6 (IL-6) (22). TNF-α can induce apoptosis by activating extrinsic apoptotic pathway (23). Apoptosis has been reported to play an important role in the development of the acute kidney injury (24). It was found that myocardial preconditioning could significantly reduce renal injury and apoptosis induced by myocardial IRI. The mechanism may be related to the inhibition of endogenous and extrinsic apoptotic pathways (25). Moreover, IRI damage underlies many ocular diseases, such as glaucoma, diabetic retinopathy, and RAO (26). IRI could lead to retinal ganglion cells death, retinal morphological degeneration, loss of retinal function and eventual loss of vision (27). We speculated that the ocular arteries after PCI might also be affected by IRI, which would further cause ocular lesions.

From a hemodynamic perspective, systemic changes in blood flow status may occur after PCI. For the most part, in the cardiovascular system, blood flow is considered laminar. When blood thinning drugs are taken, the whole blood viscosity decreases, as the Reynolds number rises and the laminar flow could be disordered and converted to turbulent. Flow turbulence enhances the energy deficit in the friction type, which increases the boundary layer blockage in the vessels and generates heat and increases the internal energy that affects the reduction of the biofluid/blood-heat-capacity-ratio. In addition, turbulence enhances the perfusion pressure necessary to push blood flow (28). Routine administration of blood thinning medications after PCI increases the risk of obstruction of internal flow due to enhanced boundary layer blockage caused by turbulence. In this study, we found that regardless of the length of time, the OA pressure of the disease groups was higher, and the mass flow ratio were lower than that of the control group. It further suggests that there are systemic changes in blood flow status after PCI. This provides a new perspective on the causes of ocular diseases after PCI.

The effect of ACS on retrobulbar blood flow should not be ignored. Consistent with our previous study (10), the blood flow velocity of the initial OA in patients with ACS after PCI was lower than that in healthy controls. However, this study further found that OA blood flow velocity of patients with ACS after PCI within 3 months was slower than the blood flow velocity of patients with ACS more than 3 months after PCI. Unfortunately, retrobulbar blood flow data after PCI are lacking. Previous studies focused on the correlation between retinal blood vessel morphology and ischemic heart disease, and the flow velocity data were few and most of them were measured using color Doppler imaging. Moreover, there is a lack of long-term observational data on retrobulbar blood flow status in patients with ACS. The cardiac index is calculated by dividing the volume of blood pumped by the heart by the surface area of the body. The higher the cardiac index, the more blood ejection of the heart and the better the heart function. We also collected statistics on the cardiac index of all patients, and the cardiac index of all patients with ACS was lower than the normal value, and the Group1 had a lower cardiac index. As mentioned above, the OA originates from the ICA and receives blood from the ICA. The Group1 in this study was still in the acute stage of the disease course, and poor cardiac pumping function may be one of the reasons for the slow flow velocity of the OA. Similarly, the OA of patients with ACS after PCI < 3 months had relatively low WSS. WSS is an important hydromechanical index related to many physiological and pathological phenomena in the cardiovascular system. There was growing evidence that atherosclerotic lesions preferentially originate in areas of flow disturbance associated with low WSS (29). In addition, low WSS accelerates endothelial turnover, leading to increased lipid uptake and promoting the formation of plaque necrotic cores (30). Moreover, the principal factor for plaque creation is time reaction between molecules and surface. Slow blood flow increases the retention time of blood through the artery, which increases the likelihood that blood particles will react with the vessel wall (31). In this study, we analyzed OA morphological data for all participants. There was no significant difference in the initial diameter of the OA, however, a larger angle was found in the OA of patients with ACS more than 3 months after PCI. This suggests that although the diameter of the OA has not changed significantly, the low flow velocity and low WSS may still affect the OA morphology (32). This suggests that more attention should be paid to the ocular condition of patients after PCI, because they may be more prone to arteriosclerosis or ocular vascular obstructive diseases.

In the correlation analysis, we found that the hemodynamic parameters of the OA correlated with some important markers of cardiac stress. Cardiac troponin T (cTnT) is considered as a marker of myocardial apoptosis and necrosis (33). The alternative mechanisms of cTnT release include increased myocardial stress due to stress and increased volumetric load. In addition, small increases in cTnT levels are associated with endothelial dysfunction and small vessel disease, not just myocardial damage (34). This suggests that cTnT may also be related to intracranial microvascular system lesions. NT-proBNP is a neurohormone mainly synthesized and secreted by ventricular myocardium, which is considered as a dynamic marker of cardiac stress (35). NT-proBNP is also associated with the regulation of retinal epithelial cells and glial cells and the function of retinal microvascular injury (36). Lower levels of NT-proBNP are associated with early microvascular changes, including loss of endothelial integrity (37), hemodynamic changes, and decreased coronary and cerebral microvascular density, which increase the risk of intracranial vascular disease (38).

This study has some limitations. ACS is an acute disease, and the first consideration the cardiologist in clinical practice is to save the life of patients. Therefore, our CTA examination was performed after the condition was stable. Moreover, the thickness of CTA scanning layer limits the accuracy of reconstruction. Finally, due to the lack of relevant research data, we set the same boundary conditions for all groups. Therefore, further research is needed to improve the integrity of these results.

## Conclusions

This study provides evidence that the OA blood flow velocity of patients with ACS after PCI initially slowed down, which increased the risk of plaque formation, and then showed an increasing trend. Moreover, there was a correlation between OA hemodynamic parameters and clinical indexes related to cardiac stress. In addition to the improvement of systemic and ocular blood perfusion after PCI, ocular vessels may be affected by IRI and blood flow status changes, resulting in ocular lesions. More attention should be paid to the ocular condition of patients after PCI. This study provides a new perspective for the pathogenesis of ocular diseases after PCI, which needs to be further explored.

## Data availability statement

The original contributions presented in the study are included in the article/supplementary material, further inquiries can be directed to the corresponding authors.

## Ethics statement

The studies involving humans were approved by the Local Ethics Committee of the Beijing Friendship Hospital. The studies were conducted in accordance with the local legislation and institutional requirements. The participants provided their written informed consent to participate in this study.

## Author contributions

W-lL: Data curation, Formal analysis, Investigation, Methodology, Writing – original draft. L-tW: Conceptualization, Data curation, Formal analysis, Investigation, Methodology, Writing – original draft, Writing – review & editing. J-lW: Conceptualization, Formal analysis, Funding acquisition, Investigation, Methodology, Resources, Supervision, Writing – review & editing. JS: Conceptualization, Formal analysis, Investigation, Methodology, Writing – original draft. X-rC: Data curation, Investigation, Methodology, Writing – original draft. Z-hZ: Investigation, Writing – original draft. J-xG: Investigation, Writing – original draft. Y-lW: Conceptualization, Funding acquisition, Methodology, Project administration, Resources, Supervision, Writing – review & editing. Z-yM: Conceptualization, Formal analysis, Project administration, Resources, Supervision, Writing – review & editing.
